# Determination of fluorine distribution in shark teeth by laser-induced breakdown spectroscopy

**DOI:** 10.1093/mtomcs/mfac050

**Published:** 2022-07-05

**Authors:** Benjamin T Manard, Christopher J Hintz, C Derrick Quarles, William Burns, N Alex Zirakparvar, Daniel R Dunlap, Toya Beiswenger, Alicia M Cruz-Uribe, Joseph A Petrus, Cole R Hexel

**Affiliations:** Chemical Sciences Division, Oak Ridge National Laboratory, Oak Ridge, TN, USA; Marine and Environmental Sciences, Savannah State University, Savannah, GA, USA; Elemental Scientific, Inc., Omaha, NE, USA; Marine and Environmental Sciences, Savannah State University, Savannah, GA, USA; Chemical Sciences Division, Oak Ridge National Laboratory, Oak Ridge, TN, USA; Chemical Sciences Division, Oak Ridge National Laboratory, Oak Ridge, TN, USA; Nuclear Nonproliferation Division, Oak Ridge National Laboratory, Oak Ridge, TN, USA; School of Earth and Climate Sciences, University of Maine, Orono, ME, USA; Elemental Scientific Lasers, Bozeman, MT, USA; Chemical Sciences Division, Oak Ridge National Laboratory, Oak Ridge, TN, USA

**Keywords:** fluorine, laser-induced breakdown spectroscopy (LIBS), mapping, shark teeth

## Abstract

Quantifying the chemical composition of fast-growing hard tissues in the environment can shed valuable information in terms of understanding ecosystems both prehistoric and current. Changes in chemical composition can be correlated with environmental conditions and can provide information about the organism's life. Sharks can lose 0.1 to 1.1 teeth/day, depending on species, which offers a unique opportunity to record environmental changes over a short duration of time. Shark teeth contain a biomineral phase that is made up of fluorapatite [Ca_5_(PO_4_)_3_F], and the F distribution within the tooth can be correlated to tooth hardness. Typically, this is determined by bulk acid digestion, energy-dispersive X-ray spectroscopy (EDS), or wavelength-dispersive spectroscopy. Here we present laser-induced breakdown spectroscopy (LIBS) as an alternative and faster approach for determining F distribution within shark teeth. Using a two-volume laser ablation chamber (TwoVol3) with innovative embedded collection optics for LIBS, shark teeth were investigated from sand tiger (Carcharias Taurus), tiger (Galeocerdo Cuvier), and hammerhead sharks (Sphyrnidae). Fluorine distribution was mapped using the CaF 603 nm band (CaF, Β ^2^Σ^+^ → X ^2^Σ^+^) and quantified using apatite reference materials. In addition, F measurements were cross referenced with EDS analyses to validate the findings. Distributions of F (603 nm), Na (589 nm), and H (656 nm) within the tooth correlate well with the expected biomineral composition and expected tooth hardness. This rapid methodology could transform the current means of determining F distribution, particularly when large sample specimens (350 mm^2^, presented here) and large quantities of specimens are of interest.

## Graphical Abstract

**Graphical Abstract:**
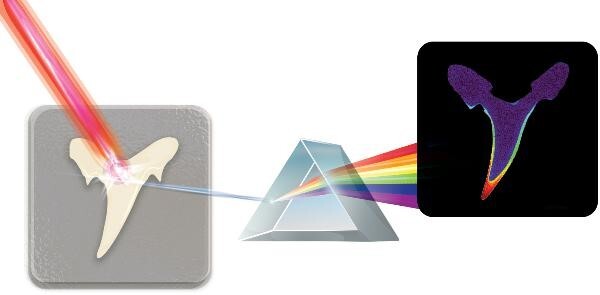
Utilization of laser induced breakdown spectroscopy for the determination of fluorine distribution in sharks teeth.

## Introduction

Apatite (calcium phosphate mineral) exhibits wide biological adaptability making it useful in a variety of biological processes, such as the construction of shells and scales, skeletal structure, and tusks and antlers.^1^^,^^2^ Apatite biominerals, or bioapatite, always exist in association with organic matrices.^2^ Historically, analysis of bulk elemental composition of whole hard tissues has limitations in the precision and the spatial distribution of these measurements has hindered impactful interpretations. Isotopic compositions of bioapatite are dependent on ecology, climate, and geology; evidence of many different factors at the time of mineralization, including environment, nutrition, and health, can be gleaned from elemental substitution into the parent mineral matrix.^3^

In natural environments the chemical compositions of fast-growing hard tissues are often changing in response to biology and chemistry in these ecosystems. Analysis of the hard tissues from modern and prehistoric species can yield an understanding of the environmental variations the organism experienced during its life. Few animals create hard tissues that are naturally discarded throughout their life. However, selachimorph elasmobranchs (i.e. sharks) replace their teeth regularly and can lose thousands over their lifetime. This creates a record of the environmental conditions over relatively discrete time bands (4–10 weeks) throughout the shark's life. Sharks have high fluoride content in their teeth, close to stoichiometric fluorapatite in the enameloid; fluoride in the enamel is higher than in the dentine layer, which is closer to a fluorohydroxyapatite.^4^

Shark teeth can be separated into two morphological groups. Orthodont teeth retain a prominent pulp cavity, whereas osteodont teeth are fully formed teeth without a pulp cavity.^5^ For both histotypes, mineralization of the osteodentine root is in the same relative location. In osteodont teeth, the region where the pulp cavity is would be filled with osteodentine^6^ following the mineralization of the root. This suggests that odontoblasts produce osteodentine in the pulp cavity after root formation. Further, osteodont teeth appear to contain different distributions of osteo- and orthodentine; specifically, osteodonts completely lack orthodentine.^7^ Beyond internal morphology and biolaminate composition, there are differences observed in the two histotypes attributed to tooth development and replacement rates. Sand tiger sharks (osteodont) replace teeth at a rate of 1.06 teeth/day, which is faster than 0.13 teeth/day reported for orthodont teeth (tiger shark, hammerhead, etc.).^8^^,^^9^ The replacement rate is related to tooth morphology, which in turn is related to target prey species for the shark; thus, diet may directly impact tooth replacement rate and have a relationship to tooth histotype.^7^

Shark teeth contain fluorapatite [Ca_5_(PO_4_)_3_F] as a biomineral phase that can contain carbonate as a phosphate substitution and hydroxide as a fluoride substitution. The presence of F in the enameloid is important as it provides structure to the tooth. Even though the P–O bonds are stronger than the Ca–F bonds, without the F for the Ca to bond to, the tooth becomes structurally weaker.^10^ Tooth hardness and Young's modulus are positively correlated with calcium and phosphate content, whereas sodium is negatively correlated.^11^^,^^12^ Overall, hardness and Young's modulus in shark teeth are very similar to those of other vertebrates, including humans.^5^ This is intriguing since fluorapatite is generally harder than hydroxyapatite.^11^ The enameloid of osteodont teeth and orthodont teeth have the same hardness and Young's modulus, though there are differences within the osteodentine and orthodentine. It is believed this effect is a function of chemical composition and microstructure.^5^

Overall, shark teeth have an optimized structure for the different functional purposes, like cutting or grasping, but the chemical composition is very similar for tooth morphological types. Since the use of fluorapatite as a biomineral does not result in a higher hardness of the tooth, it must serve a different but currently unknown purpose.^11^ For teeth that draw, hold, and puncture (e.g. sand tigers) most of the stress is focused on the top of the tooth, near the root. However, there will also be stress located along the cutting/puncturing edges.^5^ It is possible that the purpose of high concentrations of fluorine along the edge of the tooth is to retain hard, sharp cutting edges. Additional analytical data sets are needed to quantify F content as well as spatial distribution to explain the biologic/ecologic drivers and functional purpose of the F behavior in shark teeth. Research has been dedicated to performing F analysis of sharks teeth by various techniques including energy-dispersive X-ray spectroscopy (EDS),^10^ wavelength-dispersive spectroscopy (WDS),^13^ and ion-selective potentiometry.^11^ Most commonly, EDS and WDS serve as useful techniques for elemental mapping, whereas ion-selective potentiometry would require a sample dissolution prior to analysis. An alternative technique, laser-induced breakdown spectroscopy (LIBS), is proposed here.

In LIBS analysis, a short-lived laser pulse is focused onto the sample surface in which a laser-induced plasma is generated. Within this plasma, a finite amount of material (fg-ng's) is vaporized from the sample surface, and subsequently excited/ionized. The emission of the plasma (generated photons) can be captured via collection optics, separated by wavelength via a spectrometer, and detected with a respective detector (e.g. charged coupled device).^14^ This technique, coupled with the ability to raster samples of interest, allows for the ability to construct elemental maps (i.e. LIBS imaging).^15–17^ Since the first utilization of LIBS for F analysis by Cremers *et al.,*^18^ the technique has shown promising results for the detection of F in a variety of applications.^19–21^ More recently, the ability to raster the sample and generate F distribution maps has been achieved.^21^^,^^22^ Quarles *et al.* utilized the F 685.6 nm emission line to map the F distribution in bastnaesite, ultimately detecting 5–7 wt%.^21^ Weiss *et al.* more recently demonstrated the ability to map a molecular band (CaF, Β ^2^Σ^+^ → X ^2^Σ^+^).^22^ The ability to detect F is quite challenging due to its low excitation efficiency. Utilizing a CaF molecular band has been recently reviewed by Gaft *et al.,*^23^ and could be the most promising means of detecting the presence of F, assuming Ca is present. If Ca is not present, studies have explored online nebulization of a Ca solution, to generate this molecular species.^24^ Presented here is the first utilization of the LIBS technique and high-speed rastering abilities to rapidly characterize the F content in shark teeth, specifically sand tiger, tiger, and hammerhead sharks. This work employs innovative optics to collect the emitted light directly at the sample surface (within a two-volume cell) and a high-speed translational stage to rapidly raster the shark teeth (e.g. largest map in this work was 19.356 × 18.100 mm). The work described herein results in high spatial resolution maps of quantified F distributions using a new laser ablation cell (TwoVol3), with extremely high throughput. In addition, laser spots of 50–150 μm and laser pulse overlapping are explored as options for improved spatial resolution.

## Experimental

### Sample preparation

Teeth were acquired through a donation of naturally discarded teeth collected at the Georgia Aquarium (Atlanta, GA, USA) by the aquarium staff. Species analysed here include sand tiger, hammerhead, and tiger shark. Shark teeth were imbedded in an epoxy mold before sectioning. Buehler (Lake Bluff, IL, USA) EpoThin2 epoxy resin and hardener were mixed per directions: it was stirred for 2 min to allow the mixture to fully saturate and to avoid “soft” molds before being poured into a silicone mold. The shark tooth was placed inside the epoxy and centered using a plastic mixing tube. Coronal sections were created by sanding the bottom of the molds to expose the tooth. A series of increasing grit wet–dry sandpapers (ca. 400 grit) removed initial material aggressively. Upon approaching target depth for the coronal section, higher grit papers (1000 grit) slowed material removal and improved surface smoothness, removing previous tooling marks. Target layers to expose in the shark tooth include the shiny-layered enameloid (SLE) and the inner enameloid and dentin. Sagittal cuts were made with a Buehler Isomet slow-spinning abrasive 102 mm diamond saw at 1.3 mm spatial intervals, with 0.3 mm sawblade kerf, across the teeth. Lastly for all sections, 5000-grit sandpaper was used for a final polishing to facilitate a consistent surface for subsequent analysis.

#### Scanning electron microscopy/energy-dispersive X-ray spectroscopy

A thermionic-emission electron gun scanning electron microscope (SEM; Hitachi SU3800, Tokyo, Japan) was utilized to collect backscattered electron (BSE) micrographs to illustrate the structure of the cross-section of a sand tiger shark tooth mounted in epoxy. The analysis was conducted at 25 kV and variable pressure at 30 Pa to help minimize charging. Copper tape was placed over the sample to make it more conductive. The entire cross-section of the tooth was imaged using the Zigzag function proprietary to Hitachi to stitch together BSE composition images at 70× magnification. The BSE images provide information about the composition, with bright regions or higher contrast corresponding to high atomic number elements and darker regions to low atomic number. This magnification was chosen to capture most of the tooth with the least number of fields to minimize acquisition time.

The SEM is coupled with an EDAX Octane Elect Super energy-dispersive X-ray spectrometer (EDS). The characteristic X-rays generated from the sample were detected by the silicon drift detector where EDS spectra and line scans are collected, allowing identification of elements and indicating the distribution of those elements within the tooth. EDS line scans were acquired to determine the change in concentration of the elements within the enamel and dentin. The elements were manually identified and used to rebuild a weight percentage line scan with a focus on fluorine concentration in the enamel versus the dentin. The sand tiger and tiger shark teeth were all analysed at the same settings with a dwell time of 100 ms, a line width of 10 μm, with a collection every 5 μm, and a frame number of 128.

### Laser-induced breakdown spectroscopy

**Fig. 1:**
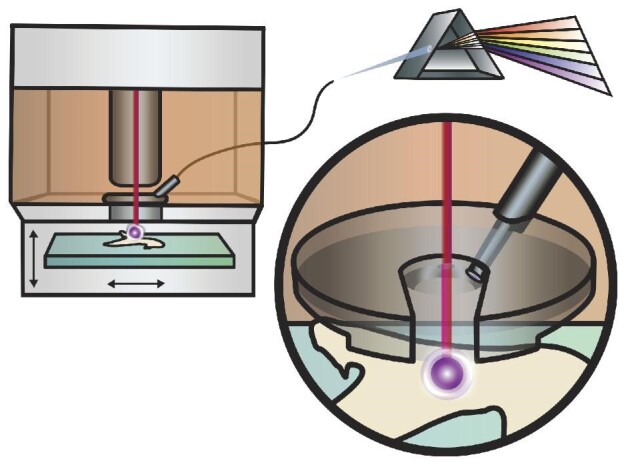
Illustration of the fiber optic position inside the TwoVol3 laser ablation cell.

An Elemental Scientific Lasers imageGEO^LIBS^ (Elemental Scientific Lasers, Bozeman, MT, USA) laser ablation system was employed for the high-speed elemental (in this case the focus is on fluorine) imaging of the shark teeth. The experimental set up is depicted in Fig. 1; the laser ablation system consists of a 193 nm excimer laser and a TwoVol3 ablation chamber. The 193 nm pulsed laser was delivered to the sample through an XYR beam aperture (this work utilized square spots 50–150 μm in width) for enhanced spatial resolution. Typical LIBS mapping through this manuscript utilized 100% laser energy, which resulted in a beam energy density at the sample surface of 10.7 J cm^–2^ at 20 Hz. For LIBS analysis, the emitted light from the laser-induced plasma was collected through an optical fiber situated directly into the “analytical cup” of the TwoVol3 chamber that is part of the imageGEO^LIBS^ platform (detailed in Fig. 1). The fiber optic was distributed into a 5-channel spectrometer (fixed grating Czerny–Turner design) with the ability to detect from 188–1099 nm utilizing a charge coupled device detector. A spectrometer delay of 0.2 μs and spectrometer integration time of 30 μs were employed. LIBS spectra were processed (atomic line identification, peak integration, and creation of 2D elemental images) in the iolite 4 software (Elemental Scientific Lasers).^25^^,^^26^ In short, the LIBS spectra were integrated such that the peak areas were determined by selecting the left and right integration windows around the targeted emission line/band. A background was subtracted from each peak utilizing a background integrated area adjacent to the peak. For example, the CaF was integrated from 599.2–607.7 nm and the selected background for subtraction was from points at 596.4 and 607.9 nm.

## Results and discussion

### Exploration of fluorine mapping in shark teeth

**Fig. 2:**
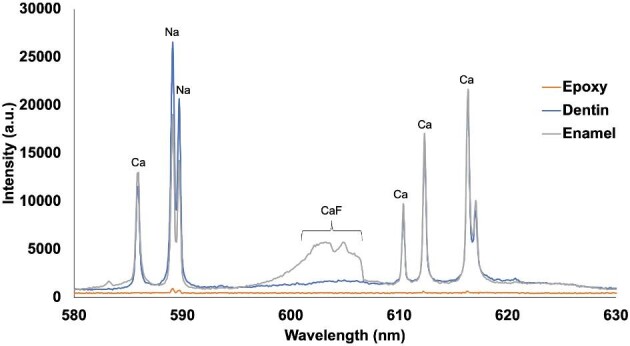
LIBS spectra (580–630 nm) showing the CaF band for epoxy used sample mounting, and the dentin/enamel of a sand tiger shark.

Initially, a sand tiger shark tooth was analysed by LIBS to determine/verify and optimize for fluorine detection in the enamel and dentin layers of the tooth. The emission of the CaF 603 nm band, A^2^Π-X^2^Σ^+^, is presented in Fig. 2. It is clearly seen that the intensity of the CaF band is ∼3× higher in the enamel layer in comparison to the dentin. This trend is expected, and has been described previously.^10^^,^^13^ Specifically, Epple *et al.*,^27^ reported that extant shark teeth (the specimen analysed in this study) have F present in the enameloid only, whereas extinct shark teeth had F in both the enameloid and the dentin. The atomic F line at 685.6 nm was not detected, which is most likely a result of the lower laser energy output from the 193 nm laser (as compared to previously published work using 266 nm lasers). The laser energy, fluence, and spot sizes in the imageGEO^LIBS^ were optimized for laser ablation–inductively coupled plasma–mass spectrometry (LA–ICP–MS). The CaF signal was optimized using 1 L min^–1^ He gas to remove atmospheric air from the cell (requirement to detect O and H in the samples) and to optimize conditions for LIBS and LA–ICP–MS data to be collected simultaneously in the future. The spectrometer delay was found to be optimal at 0.2 μs and was determined by monitoring the responses for CaF 603 nm, H 656 nm, and O 777 nm.

**Fig. 3:**
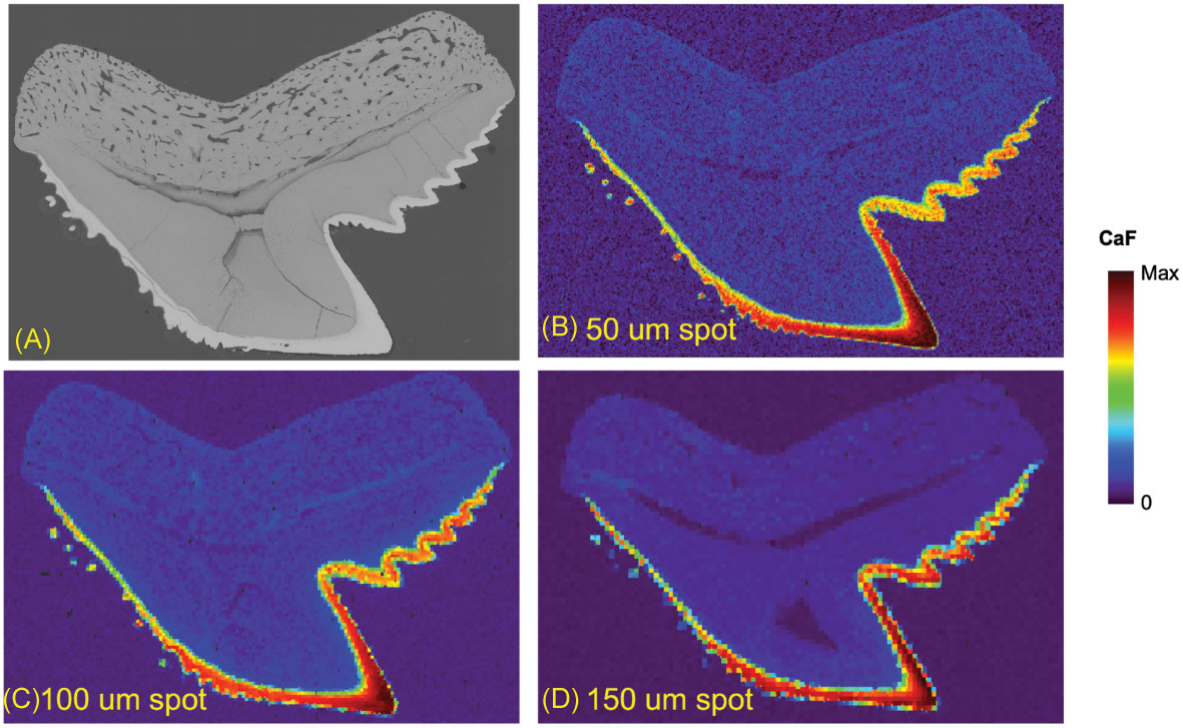
Comparison of CaF distribution in a tiger shark tooth using varying spot sizes of 50 μm (**B**), 100 μm (**C**), and 150 μm (**D**).

**Fig. 4:**
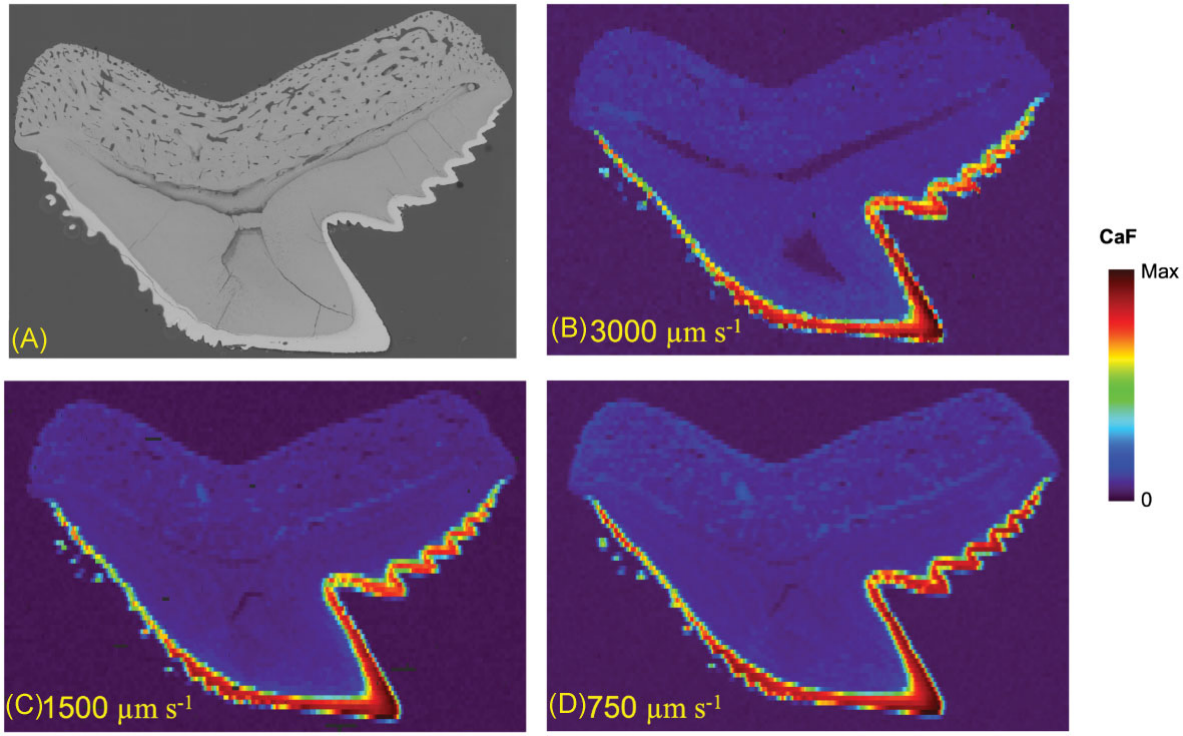
Comparison of CaF distribution in a tiger shark tooth using different stage scan speeds of 3000 μm s^–1^ (**B**), 2000 μm s^–1^ (**C**), and 1000 μm s^–1^ (**D**).

After signal optimization, a tiger shark tooth was analysed under different laser sampling conditions to determine the best parameters for comparing the three different shark species. Laser spot sizes of 50, 100, and 150 μm (square spots were used in all experiments, e.g. 50 μm is 50 × 50 μm) were used to map the distribution of CaF (Fig. 3). Raster “image” mode was used for these maps in such that no overlap in laser shots occurred in the *x*- or *y-*axis. The analysis of this tiger shark was performed over an area of 16.273 × 11.400 mm. With the respective laser spot sizes of the 150, 100, and 50 μm, this resulted in 8208, 18 582, and 73 676 LIBS spectra/sample map, respectively. The 150 μm spot size resulted in much higher intensities (400 000 a.u. max signal) than the 100 μm (150 000 a.u. max signal) and 50 μm (30 000 a.u. max signal) CaF maps. However, the resolution is improved greatly with the 50 μm CaF map compared to the 100 and 150 μm maps. In an effort to maximize signal and obtain higher resolution, the 150 μm measurements were repeated using different stage scan speeds, resulting in increased overlap of laser shots at slower speeds. Figure 4 displays the CaF distribution for three stages speeds: 3000, 2000, and 1000 μm s^–1^. Improvement in the CaF distribution can be noticed in the enamel of the tooth as more laser shots overlap, but the resolution does not reach the same quality as the 50 μm map. Therefore, it was determined that the 50 μm laser spot size was the best option for the CaF distribution maps in this study.

### Quantification of fluorine in shark teeth

**Fig. 5:**
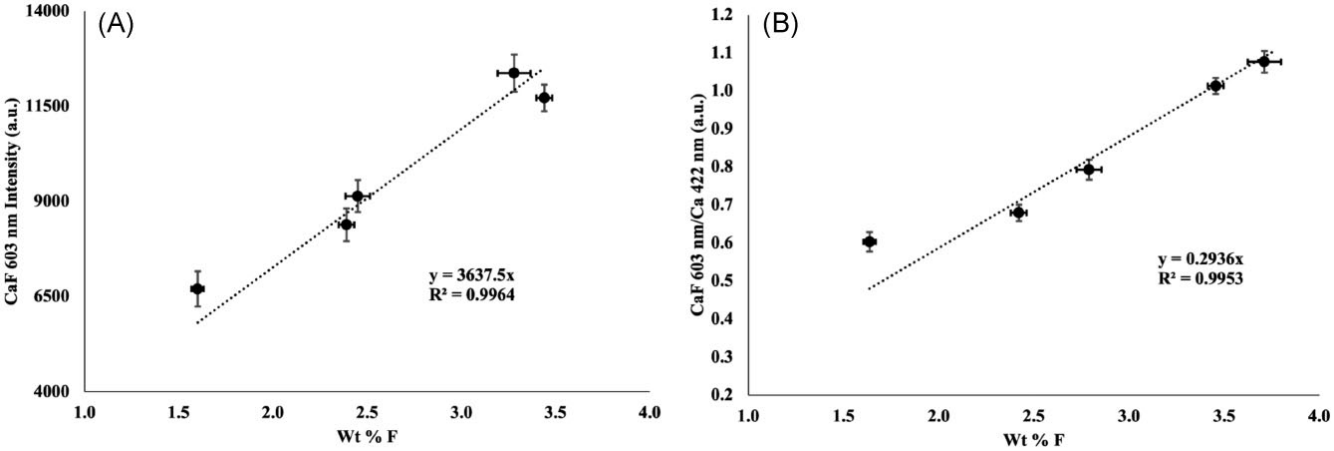
CaF and CaF/Ca response curves for the 50 μm mapping method. Intensity standard deviations (*n* = 30) and concentration standard deviations reported from work by McCubbin *et al*.^30^

Ideally, matrix-matched reference materials (RMs) are necessary for quantification in LIBS-based analysis.^28^ Here, to match the bioapatite composition of the shark teeth, five apatite samples with varying F content previously utilized by McCubbin *et al.*^29^^,^^30^ were mounted, polished, and measured by LIBS. These apatite RMs include AP003, AP004, AP005, AP018, and AP023, reported in McCubbin *et al.*,^30^ and have F contents ranging from ∼1.5 to 4 wt%. The same mapping conditions were applied, such that any matrix effects were considered. The RMs were analysed with 50, 100, and 150 μm spot sizes (as done for the LIBS imaging), and their respective calibration curve responses can be found in Table 1. These analyses were taken such that 30 laser pulses were collected. The 30 individual analyses were integrated, and the respective average and standard deviation was utilized for the generation of the external calibration curve. The calibration curve for the 50 μm laser spot is shown in Fig. 5A. Additionally, to normalize for the Ca that could be present in the RM, and subsequently the shark tooth, the CaF molecular band was ratioed to the Ca intensity, at 422 nm, and plotted in Fig. 5B. It should be noted that the Ca content in the RMs was 54.7 ± 0.6 wt%. This normalization helps demonstrate the contribution of the F species to the CaF molecular band.

**Table 1.** CaF response curves and linearity for the 50, 100, and 150 μm laser spot calibration

Spot size	CaF response curve	Standard error of slope	Linearity (*R*^2^)
50 μm	*y* = 3638*x*	109	0.9964
100 μm	*y* = 25065*x*	845	0.9955
150 μm	*y* = 70616*x*	3000	0.9929

### Comparison of fluorine distribution in different species of shark teeth

**Fig. 6:**
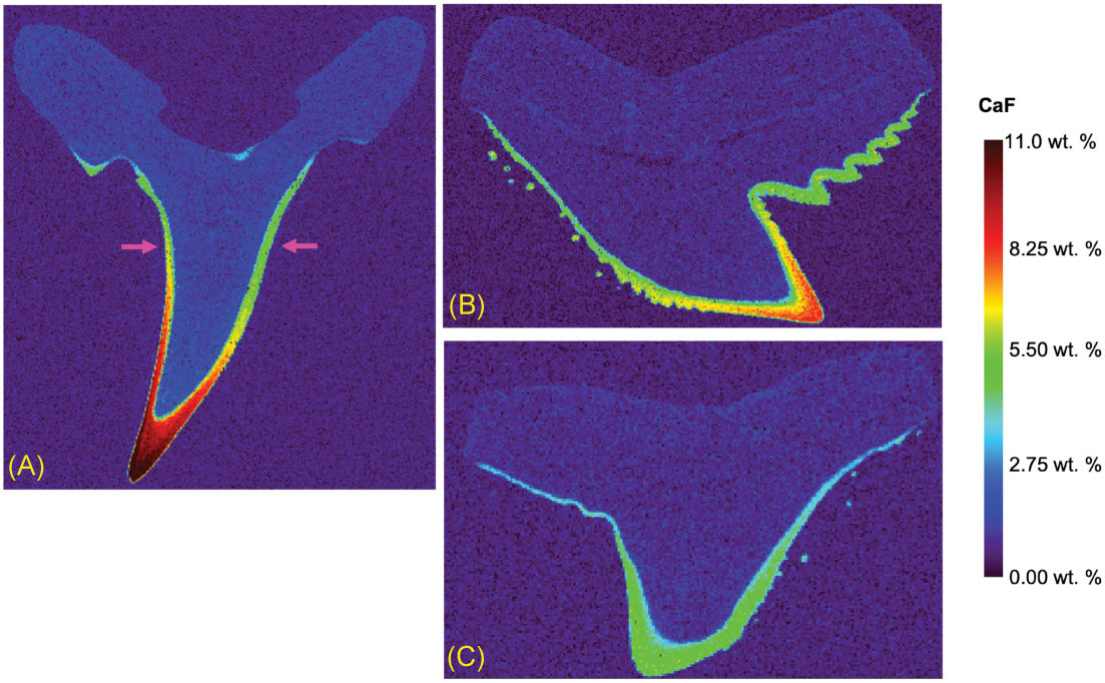
CaF distribution in teeth of the (**A**) sand tiger shark (with cross-section location—red dashed line), (**B)** tiger shark, and (**C)** hammerhead shark using the 50 μm spot method.

The F distribution in three shark species (sand tiger, tiger, and hammerhead sharks) is depicted in Fig. 6. To achieve the analysis of these three images, the sand tiger shark analysis area was 19.356 × 18.100 mm with 140 094 acquired LIBS spectra, and ∼120 min analysis time. The tiger shark analysis area was 16.273 × 11.400 mm with 73 676 acquired spectra, and ∼64 min analysis time. The hammerhead shark area was 10.732 × 7.500 mm, with 32 250 acquired spectra, and ∼28 min analysis time. It has been previously suggested that ultimately there are three layers within the enamel of a shark tooth: an internal tangled-bundle enameloid (TBE), a middle parallel-bundled enameloid (PBE), and an external SLE.^31^ Specifically, Cury *et al.*, described that the PBE and TBE have a very smooth transition between the two. However, a more distinct difference is found between the PBE + TBE and the SLE layer, which suggests that there are two “units” of the enamel: a superficial (shiny) layer and an inner layer.^4^^,^^31^ The results presented here ultimately show different regions of F within the enamel. There appears to be a higher concentration of F in the SLE layer, particularly in the sand tiger and tiger shark; additionally, there appears to be high F concentration toward the tip of the tooth in comparison to near the basal layer (top). As mentioned earlier, these findings of F concentration are not widely known, and ultimately unexplored. It is predicted that the high F content along the outer portions of the tooth is to retain hard, sharp cutting edges. Another consideration would be the difference in tooth functionality. It is interesting to note that the tooth with the higher F concentration (sand tiger) is of osteodont morphology, whereas the tiger and hammerhead shark teeth would be of orthodont morphology. Further studies would need to be explored, investigating the F content, with a larger data set of teeth from a controlled environment (such as these).

**Fig. 7:**
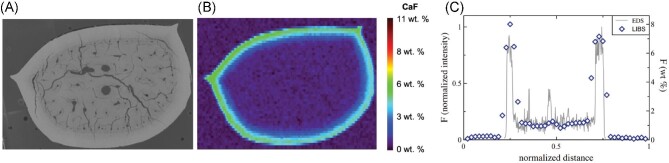
(**A**) SEM image of the horizontal cross-section of the sand tiger shark tooth, **(B**) CaF distribution (obtained by LIBS) in the horizontal cross-section of the sand tiger shark tooth, (**C**) LIBS and EDS cross-section F profile across the horizontal cross-section of the sand tiger shark tooth.

A dissection of the sand tiger shark tooth was performed along with subsequent LIBS mapping for F content. The location of the horizontal cross-section is highlighted in Fig. 6A (with arrows pointing to the section location) and its respective SEM image is in Fig. 7A. To construct the SEM image, a total of 29 fields with an overlap of 10% were collected to assemble the panorama image of the tooth. The F distribution, generated from the LIBS data, of the horizontal cross-section can be seen in Fig. 7B, which corresponds well with the vertical cross-section distribution. This sand tiger shark horizontal cross-section analysis area was 3.815 × 2.800 mm with 4312 acquired LIBS spectra, and ∼4 min analysis time. Additionally, line scans from EDS measurements were also generated and compared to the LIBS line scan, from approximately the same location. This line scan comparison can be seen in Fig. 7C. For comparison, the EDS line scan was achieved via the combination of two EDS line scans (due to spatial limitations of the SEM–EDS configuration). Each line scan took ∼2 hr (4 hr in total) compared to ∼15 s required for the LIBS line scan. This comparison between the LIBS and EDS techniques truly highlights the speed of analysis that can be achieved via the LIBS imaging, while maintaining the similar spatial profile. When comparing the data within the dentin of the two methods, the LIBS transient yielded an average concentration of 1.07 ± 0.14 and the EDS was 1.01 ± 0.81, highlighting the significant improvement in precision of the LIBS data (∼12% versus ∼80%). The Ca (422 nm) transient was also compared, such that a comparison to the CaF emission line could be made. It should be noted that the difference between the Ca within the enamel and dentin was ∼1.3× (higher in the enamel) whereas the difference in the CaF was ∼8.2× (higher in the enamel), further demonstrating the contribution from the F that is present. Ultimately, even in the small sample size (3.8 × 2.8 mm) the F distribution is clear within the enameloid region. The detailed EDS analyses, with respective line scans can be seen in Supplementary Figs. S1 and S2. The LIBS and EDS measurements correspond well regarding the F distribution within the shark teeth.

**Fig. 8:**
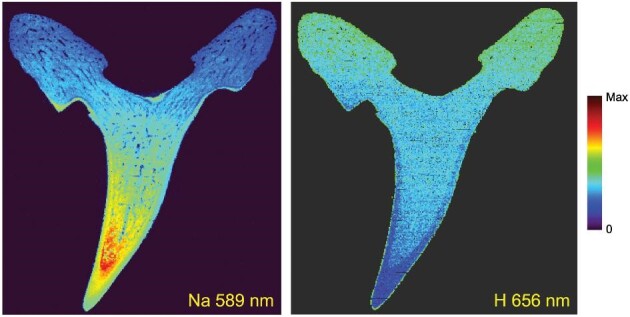
Na (589 nm) and H (656 nm) distribution in the sand tiger shark via LIBS mapping.

Lastly, since the LIBS spectra provide truly simultaneous detection of elements for each laser pulse, additional information can be correlated. The elemental distribution of Na (589 nm) and H (656 nm) are shown in Fig. 8. The F appears to replace the H (as hydroxide) in the enamel of the tooth. The H distribution is highest in the root of the tooth and decreases toward the tip of the tooth in the dentin region. The Na distribution is highest in the dentin region near the tip of the tooth and is the lowest in the root of the tooth. In addition, there is a decrease of Na found in the enamel region that corresponds well with the tooth hardness and Young's modulus. This trend has been previously described, specifically by Kocis *et al.* by use of electron microprobe, in which the dentin was Na rich.^13^

## Conclusions

A rapid (∼2 hr for a 350 mm^2^ sample map) method for the determination of F distributions throughout shark teeth is demonstrated. The uniqueness of the high-performance translation stages in the TwoVol3 laser ablation cell, coupled to a newly developed optical means of collecting the emitted light from the laser-induced plasma allows for high-speed rastering and subsequent F determination. The applicability of this technique to shark teeth allows for the spatial determination of quantified F in a variety of species from a controlled environment. Here it is determined that F concentration varies within the enameloid itself, with higher concentrations in the outer layer (external shiny layer), and that, potentially, F concentrations are higher in the osteodont morphological structures (sand tiger shark). The determination of F by LIBS offers a much faster alternative to traditional F mapping methods; e.g. a single line scan across the shark's tooth takes seconds by LIBS and up to 4 hr by EDS (for the sample size presented here). This speed of analysis will allow for future studies to be dedicated to scrutinizing larger data sets to determine the F distribution and content variation within a single species.

## Supplementary material

Supplementary data are available at *Metallomics* online.

## Supplementary Material

mfac050_Supplemental_File

## Data Availability

The data underlying this article can be shared on reasonable request to the corresponding author.
