# Supplementary material for: Determination of fluorine distribution in shark teeth by laser-induced breakdown spectroscopy
Source: Metallomics. 2022 Jul 5;14(7):mfac050. doi: 10.1093/mtomcs/mfac050 (PMC9314717; doi:10.1093/mtomcs/mfac050)
Supplement: mfac050_Supplemental_File [file mfac050_supplemental_file.docx]

**Supplementary Information**

**Determination of Fluorine Distribution in Shark Teeth by Laser Induced Breakdown Spectroscopy**

Benjamin T. Manard^1*^, Christopher J. Hintz^2^, C. Derrick Quarles Jr.^3^, William Burns^2^, N. Alex Zirakparvar^1^, Daniel Dunlap^1^, Toya Beiswenger^4^, Alicia Cruz-Uribe^5^, Joseph A. Petrus^6^, and Cole R. Hexel^1^

1. Chemical Sciences Division, Oak Ridge National Laboratory
2. Marine & Environmental Science, Savannah State University
3. Elemental Scientific, Inc.
4. Nuclear Nonproliferation Division, Oak Ridge National Laboratory
5. School of Earth and Climate Sciences, University of Maine
6. Elemental Scientific Lasers

**
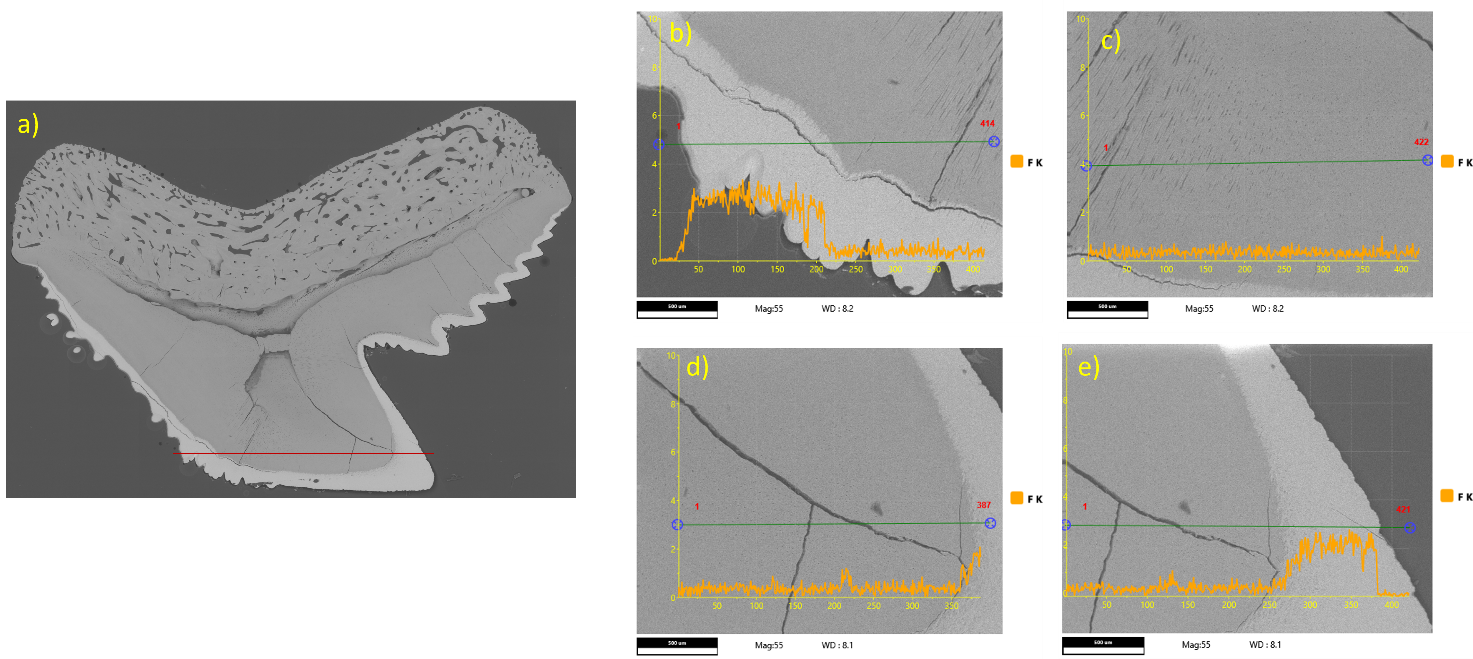
**

**Figure S1.** SEM image of tiger shark (a) tooth highlighting where the respective EDS line scan measurement was taken (red line) along with the 4 line scans (b-e) needed to make the measurement. The transients represent the fluorine distribution across the line.

**
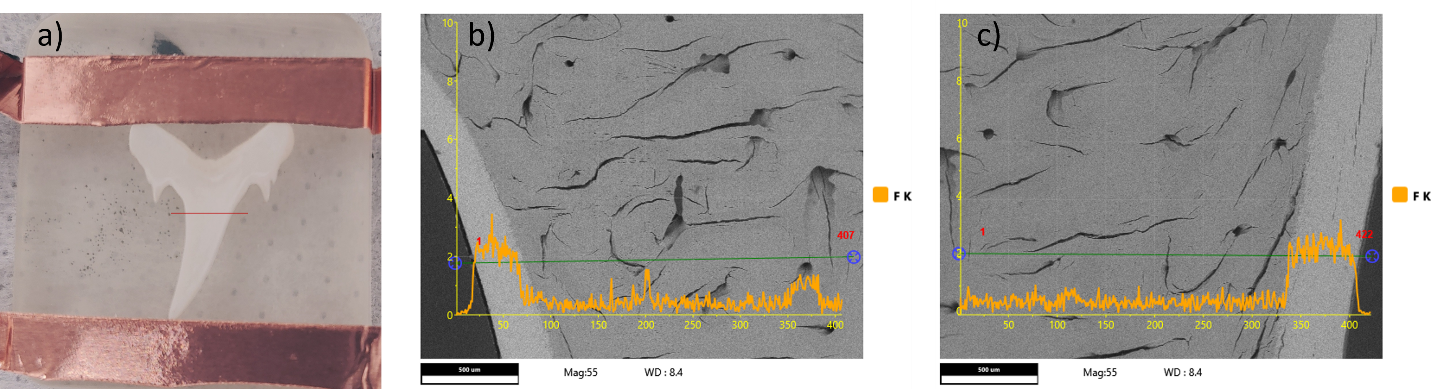
**

**Figure S2.** Photograph of sand tiger shark tooth embedded within an epoxy along with the 2 line scans (b and c) needed to make the measurement. The transients represent the fluorine distribution across the line
